# *K*_*r*_/*K*_*c*_ but not *d*_*N*_/*d*_*S*_ correlates positively with body mass in birds, raising implications for inferring lineage-specific selection

**DOI:** 10.1186/s13059-014-0542-8

**Published:** 2014-12-11

**Authors:** Claudia C Weber, Benoit Nabholz, Jonathan Romiguier, Hans Ellegren

**Affiliations:** Department of Evolutionary Biology, Evolutionary Biology Centre, Uppsala University, Norbyvägen 18 D, Uppsala, Sweden; Institut des Sciences de l’Evolution-Montpellier, UMR CNRS-UM2 5554, University Montpellier II, Montpellier, 34095 France; Department of Ecology and Evolution, University of Lausanne, Lausanne, CH-1015 Switzerland

## Abstract

**Background:**

The ratio of the rates of non-synonymous and synonymous substitution (*d*_*N*_/*d*_*S*_) is commonly used to estimate selection in coding sequences. It is often suggested that, all else being equal, *d*_*N*_/*d*_*S*_ should be lower in populations with large effective size (*N*_e_) due to increased efficacy of purifying selection. As *N*_e_ is difficult to measure directly, life history traits such as body mass, which is typically negatively associated with population size, have commonly been used as proxies in empirical tests of this hypothesis. However, evidence of whether the expected positive correlation between body mass and *d*_*N*_/*d*_*S*_ is consistently observed is conflicting.

**Results:**

Employing whole genome sequence data from 48 avian species, we assess the relationship between rates of molecular evolution and life history in birds. We find a negative correlation between *d*_*N*_/*d*_*S*_ and body mass, contrary to nearly neutral expectation. This raises the question whether the correlation might be a method artefact. We therefore in turn consider non-stationary base composition, divergence time and saturation as possible explanations, but find no clear patterns. However, in striking contrast to *d*_*N*_/*d*_*S*_, the ratio of radical to conservative amino acid substitutions (*K*_*r*_/*K*_*c*_) correlates positively with body mass.

**Conclusions:**

Our results in principle accord with the notion that non-synonymous substitutions causing radical amino acid changes are more efficiently removed by selection in large populations, consistent with nearly neutral theory. These findings have implications for the use of *d*_*N*_/*d*_*S*_ and suggest that caution is warranted when drawing conclusions about lineage-specific modes of protein evolution using this metric.

**Electronic supplementary material:**

The online version of this article (doi:10.1186/s13059-014-0542-8) contains supplementary material, which is available to authorized users.

## Background

It has long been established that different lineages evolve at heterogeneous rates [1,2] and that differences in organismal life history are reflected by rates of molecular evolution. This is readily observed in terms of lineage-specific nucleotide divergence, with small-bodied species with shorter generations tending to evolve more quickly than their larger relatives [3-10]. While this has been proposed to be a consequence of the higher number of germ cell divisions per unit time [11], the precise cause for the pattern remains unclear [12].

Another side effect of body size variation between lineages manifests in population size differences, as small species tend to have larger populations [13]. This in turn might impact the prevalence of both positive and negative selection in genome evolution relative to drift. Over long timescales, the distribution of weakly selected mutations is expected to be affected by population size, with fixation probability depending on the product of *N*_e_ and the selection coefficient, *s* [14]. Mutations with small selective effects close to the reciprocal of *N*_e_ will behave effectively neutrally [3,15-17]. Non-synonymous substitutions are on average under greater selective constraint than synonymous substitutions. As a consequence, nearly neutral theory predicts that *d*_*N*_/*d*_*S*_ should be lower in large populations [18], as relatively more slightly deleterious non-synonymous changes are removed due to increased selection efficiency when *N*_e_ is high. Consistent with this idea, pathogenic bacteria and endosymbionts have accelerated rates of protein evolution compared to their free-living relatives, as might be expected given their smaller population sizes [19,20]. Empirical studies on mammals employing body mass as a proxy for *N*_e_ in absence of actual census counts [21] present a similar pattern, with smaller-bodied species tending to have lower *d*_*N*_/*d*_*S*_ ratios than their larger counterparts [22-27] (though not all authors report a significant relationship; see [9]).

Nevertheless, this trend may not be universal. It was previously reported that there is no significant relationship between *d*_*N*_/*d*_*S*_ and life history in a data set containing 7.6 kb of coding sequence from 19 avian genes [28]. It is conceivable that this result was simply owing to insufficient statistical power, as the expected relationship between body mass and substitution rates was also not retrieved. However, more recently, Nabholz *et al*. [29] found that avian mitochondrial *d*_*N*_/*d*_*S*_ was negatively correlated with body mass. One might expect that inherent differences between inheritance, mutation rate, recombination and gene density in the mitochondria and nucleus could lead to differences in the modulation of substitution patterns by selection [30]. However, in mammals, signatures of purifying selection are reported to be congruent between nuclear and mitochondrial genes [27,31]. A complementary approach to studying the effect of population size on purifying selection is to compare island to mainland species. In principle, island endemic lineages ought to maintain life histories similar to those of their mainland relatives, while experiencing a reduction in *N*_*e*_ [32] (but see [33]). However, here the evidence is similarly equivocal for birds with both increases and decreases in *d*_*N*_/*d*_*S*_ being reported [34,35], possibly owing to lack of power [36]. Moreover, an increase in *d*_*N*_/*d*_*S*_ is predicted with increasing environmental change [37], which is expected when a species colonises an island. It therefore does not follow that an increase in *d*_*N*_/*d*_*S*_, if at all present, need necessarily be owing to a reduction in population size.

Thus, whether or not large populations generally exhibit lower *d*_*N*_/*d*_*S*_ as predicted by theory is not clear at present. To determine this, we need to study additional taxa in depth, considering possible confounding variables. One notable feature of avian genomes in particular is the strong variation in GC content between lineages associated with life history [38-40]. This may be of relevance, as estimation of *d*_*N*_/*d*_*S*_ is known to be impacted by non-stationary base composition. A degree of caution may therefore be warranted when comparing genomes that differ substantially from one another in terms of base composition.

Making use of nuclear sequences from 48 fully sequenced bird genomes, which were recently generated to resolve the phylogeny of modern birds [40], we aim to characterise the relationship between life history, *d*_*N*_/*d*_*S*_ and the efficacy of selection in birds. In doing so, we also examine to what extent method artefacts might influence our conclusions, examining in turn non-stationary base composition, divergence time, saturation and how examining different classes of amino acid change in relation to population size may help answer these questions.

## Results

### *d*_*N*_/*d*_*S*_ is elevated, not reduced, in birds with putatively larger populations

**Figure 1:**
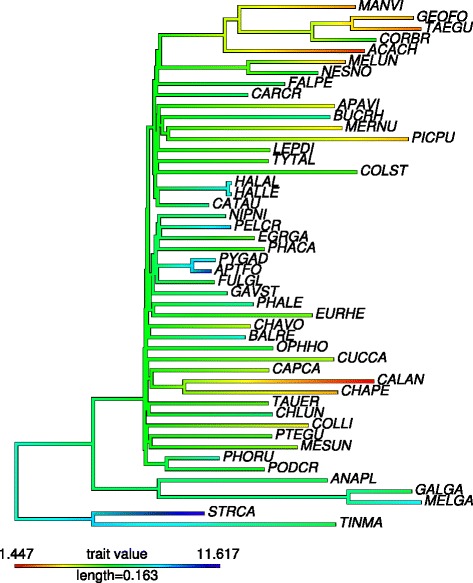
**Avian phylogeny.** Total evidence nucleotide tree from Jarvis *et al*. [40] displaying the 48 species considered in our study. Branches are coloured according to log (body mass) in grams. Full species names are given in Additional file 2.

**Figure 2:**
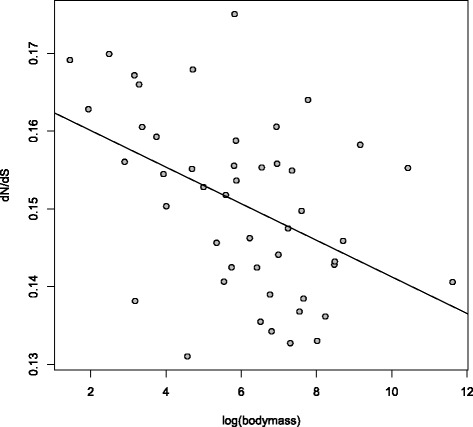
**Small-bodied species have higher** ***d***_***N***_ **/** ***d***_***S***_ **.** Body mass is significantly negatively correlated with *d*_*N*_/*d*_*S*_ (shown for the data set of 48 species).

To assess whether nuclear sequences from birds provide evidence that more efficient purifying selection in large effective populations removes a higher proportion of non-synonymous changes, we estimated lineage-specific *d*_*N*_/*d*_*S*_ for 48 species by maximum likelihood, considering 921 out of 1,185 1:1 orthologues that did not contain internal stops. We used a consensus phylogenetic tree obtained from several types of phylogenomic analyses of these 48 genomes and focused on rates in terminal branches (Figure 1). One initial observation was that variation in *d*_*N*_/*d*_*S*_ among lineages was relatively limited, in the range of 0.13 to 0.17. Our results appear to contradict the notion that more efficient protein-level selection in large populations is reflected by reduced *d*_*N*_/*d*_*S*_. Instead, body mass and *d*_*N*_/*d*_*S*_ were significantly negatively correlated (Spearman’s rank correlation: *ρ*=−0.4306,*P*=0.0027; Figure 2). To ensure the robustness of this observation, we additionally considered a data set comprising 11 kb of coding sequence from 169 avian species [41]. A similar negative correlation was seen (*ρ*=−0.3807,*P*=3.3×10^−7^; see Figure in Additional file 1). We hereafter refer to these data as the gene-rich and taxon-rich sets, respectively. Additionally, results from Coevol, which provides information on associations between traits and substitution patterns through evolutionary time using a Bayesian Monte Carlo framework rather than merely considering tip nodes [42], showed a similar negative correlation between *d*_*N*_/*d*_*S*_ and body mass for the taxon-rich set (*R*=−0.302,*pp*=0.026). This approach also corrects for phylogenetic non-independence between branches, suggesting that the result is not simply due to non-independence of the observations.

### *d*_*N*_ and *d*_*S*_ are higher in small-bodied birds

**Figure 3:**
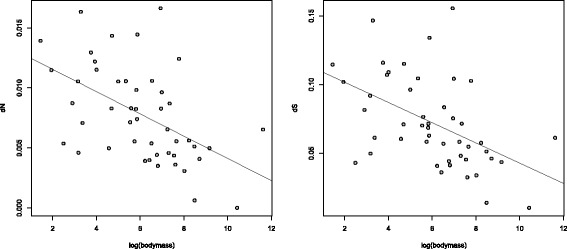
***d***_***N***_ **and** ***d***_***S***_ **negatively correlate with mass.** Small birds exhibit more rapid rates of divergence at both synonymous (*d*_*S*_) and non-synonymous (*d*_*N*_) sites (shown for the data set of 48 species).

We next surveyed synonymous and non-synonymous substitution rates and their relationships with life history individually. *d*_*S*_ behaves as predicted if small birds with short generation times evolve more rapidly, correlating negatively with body mass in both data sets (*ρ*=−0.5208,*P*=0.0002 for the gene-rich data set, Figure 3; *ρ*=−0.3015,*P*=6.8×10^−5^ for the taxon-rich data set). *d*_*N*_ was similarly negatively correlated with body mass (*ρ*=−0.5147,*P*=0.0003 for the gene-rich data set, Figure 3; *ρ*=−0.3814,*P*=3.1×10^−7^ for the taxon-rich data set). This indicates that high *d*_*N*_/*d*_*S*_ in species with putatively large populations is not due to the denominator of the ratio being smaller in absolute terms, though there must by definition be a reduction relative to *d*_*N*_. Taken at face value, these findings would seem to suggest that selection is less rather than more efficient in birds with large population sizes. It is, however, possible that the negative relationship between *d*_*N*_/*d*_*S*_ and body mass is a method artefact or is explained by another factor that covaries with life history.

### No evidence that non-stationary base composition accounts for elevated *d*_*N*_/*d*_*S*_

**Figure 4:**
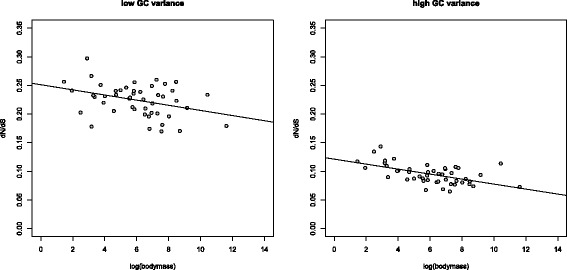
***d***_***N***_ **/** ***d***_***S***_ **vs mass for high- and low-heterogeneity orthologues.** Sequences with high and low between-species variation in GC3 show a negative correlation between *d*_*N*_/*d*_*S*_ and body mass (shown for the data set of 48 species).

In addition to the above-mentioned correlations between substitution rates and life history traits, small birds have higher GC content than large species [38]. Non-stationary composition may lead to model misspecification if not accounted for, as the underlying models assume codon frequencies to be at equilibrium. This can impact estimates of divergence and lead to false conclusions [43,44]. Considering only orthologues with low variance in GC3 content (see Materials and methods), where we would expect less impact of compositional differences on rate estimation, we observed a reduction in the strength of the negative correlation relative to the high-variance set (*ρ*=−0.3018,*P*=0.0396 for the low variance set; *ρ*=−0.5307,*P*=0.0001 for the high variance set; Figure 4). However, the sign of the correlation did not reverse. We additionally calculated the correlation between body mass and *d*_*N*_/*d*_*S*_ through time controlled for equilibrium GC content using Coevol. This did not alter the correlation coefficient (*R*=−0.302,*pp*=0.019 for the taxon-rich set). Note that median *d*_*N*_/*d*_*S*_ was lower for the high-variance subset (median 0.0939) than for the low-variance set (median 0.2301; Wilcoxon test *P*=2.2×10^−16^; Figure 4).

### Divergence time and estimation of *d*_*N*_/*d*_*S*_

Another possibility that may explain the negative relationship between *d*_*N*_/*d*_*S*_ and *N*_e_ is that there is a dependence of *d*_*N*_/*d*_*S*_ on time. When divergence times are short, the ratio may be inflated owing to artefacts that can be statistical or biological in nature and do not reflect a genuine acceleration in the evolutionary rate. Here, both divergence times and terminal branch lengths are determined by the phylogeny considered. Explanations that have been proposed for this include segregating deleterious non-synonymous polymorphisms, the non-linear dynamics of the ratio of the two variables, and model misspecification due to failure to account for amino acid preference in different protein domains [45-50]. The time required for this effect to decay has been suggested to depend on *N*_e_ [47], which could be potentially problematic for our data given that we find a positive correlation between body mass and time since divergence from the most recent ancestor (*ρ*=0.406,*P*=0.0127; calibration points with confidence intervals in the upper quintile were excluded), indicating shorter times for birds with larger populations. Meanwhile, *d*_*N*_/*d*_*S*_ is negatively correlated with divergence time, that is, *d*_*N*_/*d*_*S*_ is higher for shorter branches (*ρ*=−0.3288,*P*=0.047; note that passerines have especially short branches, see Figure 1). However, while controlling the correlation between body mass and *d*_*N*_/*d*_*S*_ for divergence time leads to a reduction in the correlation coefficient (partial Spearman’s rank correlation *β*=−0.3211,*P*=0.0480, compared to *ρ*=−0.4106, *P*=0.0122 for *d*_*N*_/*d*_*S*_ versus mass for the filtered data set), it does not altogether remove the relationship, which remains marginally significant. On the other hand, controlling the correlation between divergence time and *d*_*N*_/*d*_*S*_ for body mass has a greater impact and renders it non-significant (*β*=−0.1945,*P*=0.2476). Finally, it should be noted that our data set mainly consists of relatively divergent lineages (>90*%* have divergence times 20 to 73 million years ago), where any time dependence on *d*_*N*_/*d*_*S*_ should be limited.

### Saturation at third codon positions may impact estimation of *d*_*S*_

Although *d*_*S*_ is often used as a proxy for the mutation rate when considering the *d*_*N*_/*d*_*S*_ ratio, this idea ought to be treated with caution [51-53]. A reduction in *d*_*S*_ could be caused either by a *de facto* constraint on the rate of synonymous substitution, or methodological limitations such as saturation that lead to underestimation of the true rate. This is of particular concern for the estimation of *d*_*N*_/*d*_*S*_ as synonymous rates might be more prone to underestimation than non-synonymous rates, because non-synonymous substitutions are generally less commonly fixed.

**Figure 5:**
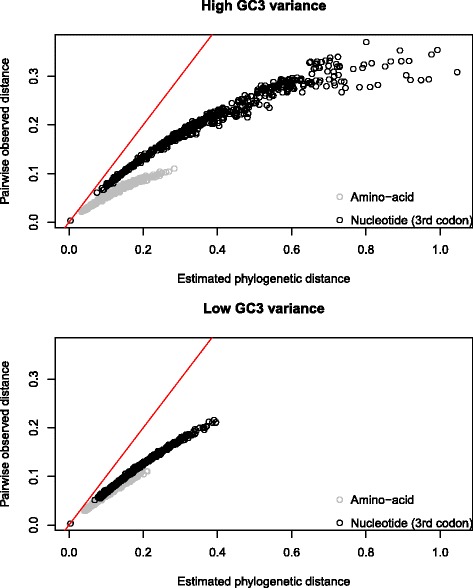
**Saturation for substitution at third sites and amino acid changes.** Uncorrected observed differences are smaller than estimated phylogenetic distances for third codon sites and amino acids. The red line denotes the relationship expected in the absence of saturation (*x*=*y*), as the phylogenetic and observed distances would be equal in this case. Divergence for third codon positions is greater than for amino acids, particularly in orthologues with high variance in GC3 between species.

To assess whether there is evidence for saturation in our data, we compared the phylogenetic distance (the sum of branch lengths between two given species) to the number of uncorrected pairwise differences for high- and low-variance sequences, as considering the full data set would not have been computationally tractable. That the uncorrected distance does not increase linearly with the corrected distance for the high-variance subset, instead remaining lower (Figure 5), indicates that there are multiple hits. As expected, divergence for third codon positions is greater than for amino acids. This implies that a degree of saturation and therefore underestimation of *d*_*S*_ relative to *d*_*N*_ might be of concern for our data. The weaker signal of saturation at third positions relative to amino acids in the lower-variance subset is consistent with the shorter branch lengths observed here (Figure 5). Constraint cannot explain the patterns we observe in the saturation plots, as it would affect both observed and phylogenetic distances. Nevertheless, the extent to which saturation affects our estimates of *d*_*N*_/*d*_*S*_ is not clear.

### Radical amino acid changes are less frequent in birds with large populations

If saturation at third sites is stronger than for amino acid substitutions and/or if many non-synonymous substitutions behave as effectively neutral, we may consider an alternative metric to assess how effectively slightly deleterious changes are purged from large bird populations. Radical amino acid changes that alter the polarity or volume of a residue are more likely to be negatively selected than conservative amino acid changes, as selective effects tend to be greater where replacements involve residues with dissimilar properties [54,55]. The ratio of radical to conservative substitutions has been suggested to be an appropriate means of testing the predictions of nearly neutral theory and overcoming saturation [29,55-57]. Here, we therefore employ *K*_*r*_/*K*_*c*_ as our metric, where *K*_*r*_ and *K*_*c*_ respectively denote radical and conservative changes.

**Figure 6:**
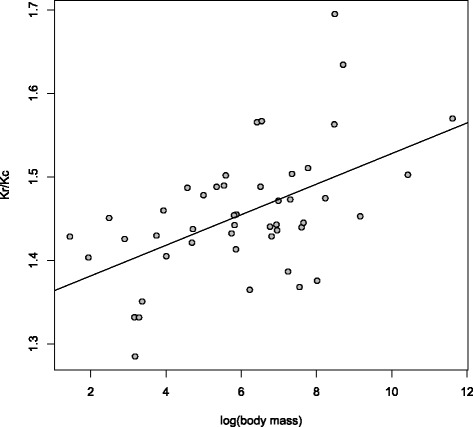
***K***_***r***_ **/** ***K***_***c***_ **correlates positively with body mass.** Large-bodied species tend to have elevated *K*_*r*_/*K*_*c*_ compared to small-bodied species, in principle consistent with less effective purifying selection (shown for the data set of 48 species).

**Figure 7:**
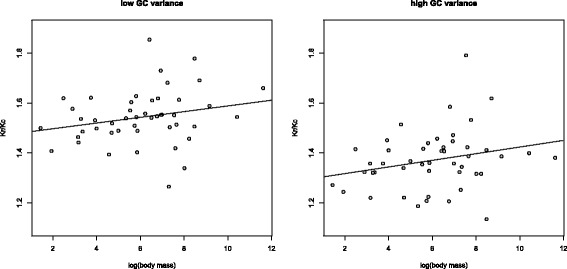
***K***_***r***_ **/** ***K***_***c***_ **correlates positively with body mass for both GC-heterogeneous and GC-homogeneous genes.** Sequences with high and low between-species variation in GC3 show a positive correlation between *K*_*r*_/*K*_*c*_ and body mass (shown for the data set of 48 species).

As expected given that *d*_*N*_ is higher in small birds, both *K*_*r*_ and *K*_*c*_ correlate negatively with body mass (*K*_*r*_: *ρ* = −0.5338,*P* = 0.0001; *K*_*c*_: *ρ* = −0.5872,*P* = 2.1 × 10^−5^) for the concatenated orthologues from the data set of 48 species. In stark contrast to *d*_*N*_/*d*_*S*_, *K*_*r*_/*K*_*c*_ is positively correlated with body mass (*ρ*=0.4998,*P*=0.0004; Figure 6), suggesting that radical changes are more frequently removed from lineages with large populations. Results from Coevol confirm the positive relationship between body mass and *K*_*r*_/*K*_*c*_ for sequences with high (*r*=0.61,*pp*=1.0) and low variance in GC3 (*r*=0.85,*pp*=1.0). It is also interesting to note that *K*_*r*_/*K*_*c*_ is somewhat reduced in the high-variance subset (median 1.3599) compared to the low-variance subset (median 1.5408; Wilcoxon test *P*=7.2×10^−12^; mapNH results; Figure 7), paralleling the differences we observed for *d*_*N*_/*d*_*S*_ (see Figure 4).

It should be noted that differences in base composition might affect the estimation of radical and conservative changes [55,56]. Given the well-characterised heterogeneity in GC content between our species, we ask whether our results are robust to control for composition. While the partial correlation for *K*_*r*_/*K*_*c*_ and mass controlling for GC3 is slightly reduced (*β*=0.3882,*P*=0.0057), the correlation for GC3 and *K*_*r*_/*K*_*c*_ controlling for mass becomes non-significant (*β*=−0.0431,*P*=0.7770 compared to *ρ*=−0.3215,*P*=0.0298). We thus find no evidence that base composition explains our observations. Note also that composition is more homogeneous between lineages in the low-variance data but this does not diminish the correlation. These results therefore support the idea that in birds radical amino acid changes are indeed more often removed from large populations than from small populations.

## Discussion

Employing a data set comprising 1,185 orthologues from 48 recently sequenced bird genomes, we examined relationships between life history and lineage-specific patterns of substitution. We found no evidence of reduced *d*_*N*_/*d*_*S*_ in birds with putatively higher effective population size, in apparent contradiction to nearly neutral theory. On the contrary, we consistently saw a negative correlation between body mass and lineage-specific *d*_*N*_/*d*_*S*_, similar to what was recently reported based on analyses of avian mitochondria [29]. This is particularly striking and not necessarily expected, given the many inherent differences between nuclear and mitochondrial sequences, as well as the fact that we were able to consider a much larger data set here. Our observations contrast with reports of a positive correlation between body mass and *d*_*N*_/*d*_*S*_ in mammals. However, considering the ratios of radical to conservative amino acid substitutions, we found a positive correlation between body size and *K*_*r*_/*K*_*c*_, meaning that lineages with putatively larger populations experience relatively fewer changes that alter the polarity and volume of a residue. That is, those differences that do occur in small-bodied birds may be less likely to disrupt protein function, which is in principle consistent with the notion that selection will more effectively purge deleterious changes from large populations. In contrast with *d*_*N*_/*d*_*S*_-based estimates, our amino acid substitution data (*K*_*r*_/*K*_*c*_) therefore appear to accord with the predictions of the nearly neutral theory. If a significant proportion of non-synonymous substitutions are conservative and behave as effectively neutral, this may obscure (expected) correlations between *d*_*N*_/*d*_*S*_ and life history. Thus, in this case, *K*_*r*_/*K*_*c*_ could potentially be a more fine-grained measure for assessing the prevalence of protein-level selection in different lineages.

While radical amino acid mutations should be subject to stronger negative selection, it has been suggested that adaptive evolution may lead to similar proportions of radical and conservative fixation [55]. Could increased rates of adaptive evolution in small birds be responsible for our observation that *d*_*N*_/*d*_*S*_, but not *K*_*r*_/*K*_*c*_, increases with decreasing body mass? Given a high proportion of effectively positively selected mutations, we might predict that the rate of fixation will increase with population size [16]. However, to affect the genome-wide average substantially, positive selection would need to be common, which is unlikely to be the case in vertebrate species with modest population sizes. The absence of high-resolution diversity data limits our ability to quantify directly the prevalence of adaptive non-synonymous substitutions in our study species. Although a past survey of chicken and zebra finch divergence and diversity data estimated the frequency of amino acid changes driven to fixation by positive selection (*α*) to be around 20% [58], this value did not differ significantly from zero. Further, simulations indicate that the influence of *N*_e_ on the proportion of adaptive amino acid changes is limited, impacting mainly populations under 10,000 [37]. Since birds typically have larger *N*_e_ than this, we might not necessarily expect differences in *N*_e_ to lead to adaptive changes being more common in smaller-bodied species. This prediction is for instance reflected in the similar percentage of fixations driven by positive selection in *Drosophila miranda* and *D. melanogaster* despite a fivefold difference in population size [59].

There are several conceivable explanations for the discrepancy between our results for the relationship between *d*_*N*_/*d*_*S*_ and *N*_e_ and theoretical expectations. One possibility is that body mass is a poor proxy for population size in birds [60], but it is not clear how this alone could lead to a reversal in the sign of the correlation, though it could in principle introduce noise. Moreover, the fact that we correlated body size of a single extant species with substitution rates reflecting evolutionary processes in multiple ancestors over significant periods of time naturally means that strong relationships cannot be expected. Another is that there was limited variation in *d*_*N*_/*d*_*S*_ (0.13 to 0.17), again weakening the signal in the data. Further, there was some evidence that third sites could be moderately saturated, indicating that we tend to underestimate synonymous changes for greater divergences, such as those observed in small-bodied bird lineages. How much of the variation this might explain is not clear, and divergence appears somewhat low for saturation alone to have a large impact. Given significant constraint on fourfold degenerate sites in birds [61], a reduction in *d*_*S*_ could also be caused by selection on silent sites. However, there is currently no evidence for a correspondence between constraint and population size [51,61]. Interestingly, we find that species *d*_*N*_/*d*_*S*_ and *d*_*S*_ are positively correlated (*ρ*=0.535,*P*=0.0001), counter to what one might expect given that *d*_*S*_ is the denominator of *d*_*N*_/*d*_*S*_. This could either indicate a bias in rate estimation or merely be an artefact of the correlations between rates and life history. It is possible that multiple factors work together to produce the pattern observed. Indeed, restricting analyses to orthologues conserved across multiple species can in itself reverse already weak correlations between genomic parameters [62].

Further, non-stationary GC content can affect estimation of substitution rates, but we detect no clear evidence for this. Given the well-established role of GC-biased gene conversion (gBGC), in driving heterogeneity in avian base composition [38,63,64], it could also impact substitution rates. gBGC is associated with the rate of meiotic recombination and leads to the preferential fixation of GC over AT alleles [65-67]. *d*_*N*_ in particular has been suggested to increase near mammalian recombination hotspots in the absence of positive selection as a result [68-72]. Since small-bodied bird species tend to have increased GC content [38], it is tempting to speculate that *d*_*N*_/*d*_*S*_ could be inflated in these lineages. In mammals, correlations between body mass and *d*_*N*_/*d*_*S*_ are partly masked by the effects of gBGC overcoming weak selection [9]. However, the impact of gBGC on global *d*_*N*_/*d*_*S*_ is difficult to assess conclusively given that we do not have relevant information on rates of recombination for the majority of our study species. This should be further investigated once detailed estimates of recombination rates become available. Interestingly, no AT → GC bias is seen in rapidly diverging sequences between chicken and zebra finch [73].

An additional issue that could affect the estimation of *d*_*N*_/*d*_*S*_ is the quality of the sequence alignments from which rates are estimated. In principle, if aligned sequences from small-bodied birds were more prone to false positive homology calls, spurious non-synonymous substitutions may be inferred, resulting in a potentially upward-biased *d*_*N*_/*d*_*S*_. While theoretically possible [74], removing the impact of alignment uncertainty on inferred substitution rates is currently prohibitively computationally costly. Several authors have previously discussed the impact of aligner choice on the rate of false positive inference of positive selection [75-77], and report that certain algorithms perform better than others. We emphasise that the first pass of alignments for the data set of 48 species was performed using SATé+PRANK (see Materials and methods), and that the class of aligners that PRANK belongs to appears less prone to false positives than others [75-77]. As such, our approach ought to be as robust as is currently feasible for a data set of this size. To address these limitations conclusively, comprehensive studies on the impact of sequence divergence on alignment uncertainty as well as further advances in alignment and rate estimation methods will be needed.

We finally note that an alternative explanation might be that the discrepancy between *K*_*r*_/*K*_*c*_ and *d*_*N*_/*d*_*S*_ is not merely owing to methodological artefacts relating to measuring *d*_*N*_/*d*_*S*_ accurately but that our naive model of how substitution rates ought to relate to population size is incomplete. The range of *N*_e_ across which nearly neutral dynamics are expected to hold depends on the distribution of selective effects that is assumed [16]. Some models propose that the distribution of selection coefficients for mutants depends on current fitness, impacting the rate of acceptance of slightly deleterious mutations [78-80]. Accordingly, it has been suggested that dependence of *d*_*N*_/*d*_*S*_ on *N*_e_ may be weak [78], with changes in population size rather than population size *per se* modulating *d*_*N*_/*d*_*S*_ and both expansions and contractions leading to increases in the ratio [33,79]. The rate of diversification appears to correlate positively with the rate of molecular evolution in bird but not mammalian lineages [28,81], tempting speculation that rapidly evolving birds are especially prone to frequent population size fluctuations. However, to explain our observations, under the size fluctuation model *K*_*r*_/*K*_*c*_ would have to be relatively less sensitive than *d*_*N*_/*d*_*S*_ to changes in *N*_e_ and more sensitive to *N*_e_ itself.

## Conclusions

Although branch-specific estimates of *d*_*N*_/*d*_*S*_ show no evidence for more efficient selection in large bird populations, *K*_*r*_/*K*_*c*_ estimates appear to conform to the predictions of nearly neutral theory in birds, with small-bodied birds tending to have fewer radical amino acid changes. If, as one interpretation of our work suggests, *K*_*r*_/*K*_*c*_ is more robust in certain scenarios, gathering deeper insight into the dynamics of this measure will be of broad relevance for inference of protein-level selection. Further, we suggest that the role of gBGC and how the distribution of selective effects differs between different populations will need to be elucidated to determine conclusively to what extent *d*_*N*_/*d*_*S*_ is determined by population size under the nearly neutral theory of molecular evolution.

The practical implications of our observations depend partly on the precise mechanisms responsible. How, for instance, might tests for positive selection be influenced? One might imagine that an upward bias in *d*_*N*_/*d*_*S*_ within a given lineage could lead to the naive assumption that a higher proportion of coding sequences with an average *d*_*N*_/*d*_*S*_>1 indicates more frequent adaptation. How branch-site tests might be affected is difficult to predict without knowing the distribution of sites that violate our assumptions of how *d*_*N*_ and *d*_*S*_ ought to behave. It has been suggested that branch-site models may lack power when saturation is present, but are less likely to yield false positives [82]. This contrasts with the higher expected rate of false positives caused by alignment problems [75-77]. We also note that comparisons between species and comparisons of different classes of sequence within genomes are expected to be affected differently by certain artefacts. For instance, ecological shifts might affect lineage-specific rate estimates to a greater extent than gene-specific rates [16], while a constraint on *d*_*S*_ [53] could impact *d*_*N*_/*d*_*S*_ in both cases.

Overall, our observations suggest that a careful examination of potential sources of error is called for when interpreting evolutionary rate estimates, and that this must be done with the specific questions and data set in mind. Further, while we cannot presently conclude that radical and conservative rates are inherently more reliable for detecting negative selection, the fact that *d*_*N*_/*d*_*S*_ does not consider the effects of different classes of non-synonymous change suggests that it likely presents an incomplete picture of selective processes.

## Materials and methods

### Sequence alignments

#### Data for 48 genomes

Coding sequence alignments for 48 bird species (see Additional file 2) were obtained from a recent initiative to resolve the phylogeny of modern birds; see Jarvis *et al*. [40] and Zhang *et al*. [83] for a detailed description of how these data were generated. Briefly, this data set comprises 8,295 orthologous protein-coding sequences identified by propagating chicken and zebra finch annotations to the remaining species and classifying orthology by combining information from alignment statistics, reciprocal best hits and synteny. Multiple sequence alignments were generated by running SATé+PRANK followed by SATé+MAFFT on concatenated exon sequences [40]. Of 1,185 1:1 orthologues present in all species, 921 contained no internal stop codons. Concatenated alignments comprising the highest and lowest variance in GC3 from the same study were also considered [40].

#### Data for 169 species

To extend our taxon sampling, we also analysed 11,160 bp of sequence from 169 avian species, consisting of the coding sequences of the Hackett *et al*. [41] data set and two additional widely used phylogenetic markers, RAG1 and RAG2, which were downloaded from GenBank (see Additional file 3 for accession numbers). The marker sequences were translated into amino acids, aligned using MUSCLE [84] and subsequently converted back to nucleotides. These data are what we refer to as the taxon-rich set.

### Life history traits

Body mass data were extracted from the *CRC Handbook of Avian Body Masses* [85] for all available tip nodes. Where multiple entries for a given species were present, the mean value was used.

### Phylogenetic trees

For the taxon-rich data set, we used the tree of Hackett *et al*. [41]. For the 48 genomes, the total evidence nucleotide tree estimated by Jarvis *et al*. [40] was used, along with corresponding time calibration points, which we considered for our divergence time analyses.

### Sequence divergence

#### Maximum likelihood estimation

Given the difference in the sizes of the two alignment data sets, as well as in the evolutionary distances between the sampled taxa, we employed two different methods of maximum likelihood estimation. To make the analyses on the larger gene-rich data set with less dense taxon sampling tractable, we approximated branch-specific *d*_*N*_/*d*_*S*_ ratios by substitution mapping using mapNH [24,86]. We did this by fitting a homogeneous YN98 [87] model to coding sequence alignments and subsequently mapping synonymous and non-synonymous substitutions onto individual branches. This was done separately for each orthologue from the 1:1 set that did not include an internal stop, and *d*_*N*_/*d*_*S*_ was obtained by summing substitution counts prior to dividing to avoid low count numbers introducing noise. To make these numbers comparable to those from Codeml, the ratio of non-synonymous to synonymous counts was divided by 3. As the branches leading to the two eagles were too short to estimate *d*_*N*_/*d*_*S*_ reliably, we considered only *Haliaeetus albicilla*.

*d*_*N*_ and *d*_*S*_ were obtained by fixing *ω*=1 in mapNH (following the rationale presented in Yang and Nielsen [87], p. 411) and multiplying the resulting normalised substitution counts by the corresponding branch lengths. This feature is implemented in the development version of Bio++ [88], available online [89].

On the other hand, for the 11-kb taxon-rich data set, rates were estimated using Codeml [90] with lineages grouped by taxonomic order to reduce variance in *d*_*N*_/*d*_*S*_ owing to short branches. We assigned one local *d*_*N*_/*d*_*S*_ for every avian order, resulting in 53 local values (see Additional file 4 for groups). Concatenating the alignments further served to reduce noise.

The ratio of radical to conservative amino acid changes (*K*_*r*_/*K*_*c*_) for the taxon-rich data set was calculated by concatenating 1,185 1:1 orthologues, fitting a Jukes–Cantor model and mapping radical and conservative substitution counts onto the tree using mapNH. Radical changes are those that alter the polarity or volume of the residue. Here, L, I, F, M, Y, W, H, K, R, E and Q were classified as having large volumes, while Y, W, H, K, R, E, Q, T, D, N, S and C were classified as polar. Results using a WAG01 model were qualitatively similar to those calculated using the Jukes–Cantor model. Considering each orthologue individually before summing counts yielded noisy results, presumably owing to low numbers of radical amino acid substitutions in individual alignments. Overall, performance was better where a greater number of substitution counts was available, as using the full set of 8,295 orthologues yielded a slightly stronger correlation between body mass and *K*_*r*_/*K*_*c*_ than when smaller subsets were considered (*ρ*=0.513,*P*=0.0003). Due to the short eagle branches, *Haliaeetus leucocephalus* was excluded.

#### Bayesian estimation of coevolution between substitution and life history

Coevol [42] was used on subsets of the gene-rich data set to calculate *K*_*r*_/*K*_*c*_ and *d*_*N*_/*d*_*S*_. As above, the polarity and volume definition (-polvol) was used to classify amino acid changes as radical or conservative. To control the relationship between body mass and *d*_*N*_/*d*_*S*_ for equilibrium base composition, we also ran Coevol with equilibrium GC as a parameter. A more detailed description of the methods used, as well as priors and calibration points, is given in Nabholz *et al*. [29].

### Saturation analysis

From the 830 orthologues with the highest and lowest variance in GC3, 200 genes were randomly selected [40]. The pairwise divergence was computed from the number of observed differences between two sequences without correction for multiple substitutions. The phylogenetic distance (that is, the patristic distance) was obtained from the sum of branch lengths between two species, computed using a phylogenetic tree estimated by maximum-likelihood using PAML. We used a GTR+GAMMA model in baseml [90] for the third codon position data set and WAG, an empirical substitution matrix, in Codeml [90] for the protein data set.

### Statistics and data availability

Statistical analyses were performed in R. The genome data from the 48 bird species are available online [91].

## Additional files

Additional file 1
***d***_***N***_ **/** ***d***_***S***_ **versus mass for the species-rich set.** Supplementary information.

Additional file 2
**Species table.** Table of species names and abbreviations.

Additional file 3
**GenBank accession numbers.** Accession numbers for RAG1 and RAG2 sequences used in taxon-rich set.

Additional file 4
**Taxon grouping.** Groups of taxa used for Codeml *d*_*N*_/*d*_*S*_ analysis.

## Abbreviations

bp: base pair
gBGC: GC-biased gene conversion
kb: kilobase
