# Supplementary material for: Kr/Kc but not dN/dS correlates positively with body mass in birds, raising implications for inferring lineage-specific selection
Source: Genome Biol. 2014 Dec 11;15(12):542. doi: 10.1186/s13059-014-0542-8 (PMC4264323; doi:10.1186/s13059-014-0542-8)

Supplementary file 1

d_N/d_S correlates negatively with body mass ($\rho = -0.3807, p = 3.3e-07$) for an alignment from 169 avian species using the data of Hackett et al. (2008) and two additional phylogenetic markers (RAG1 and RAG2; see Additional file 3 for accession numbers).

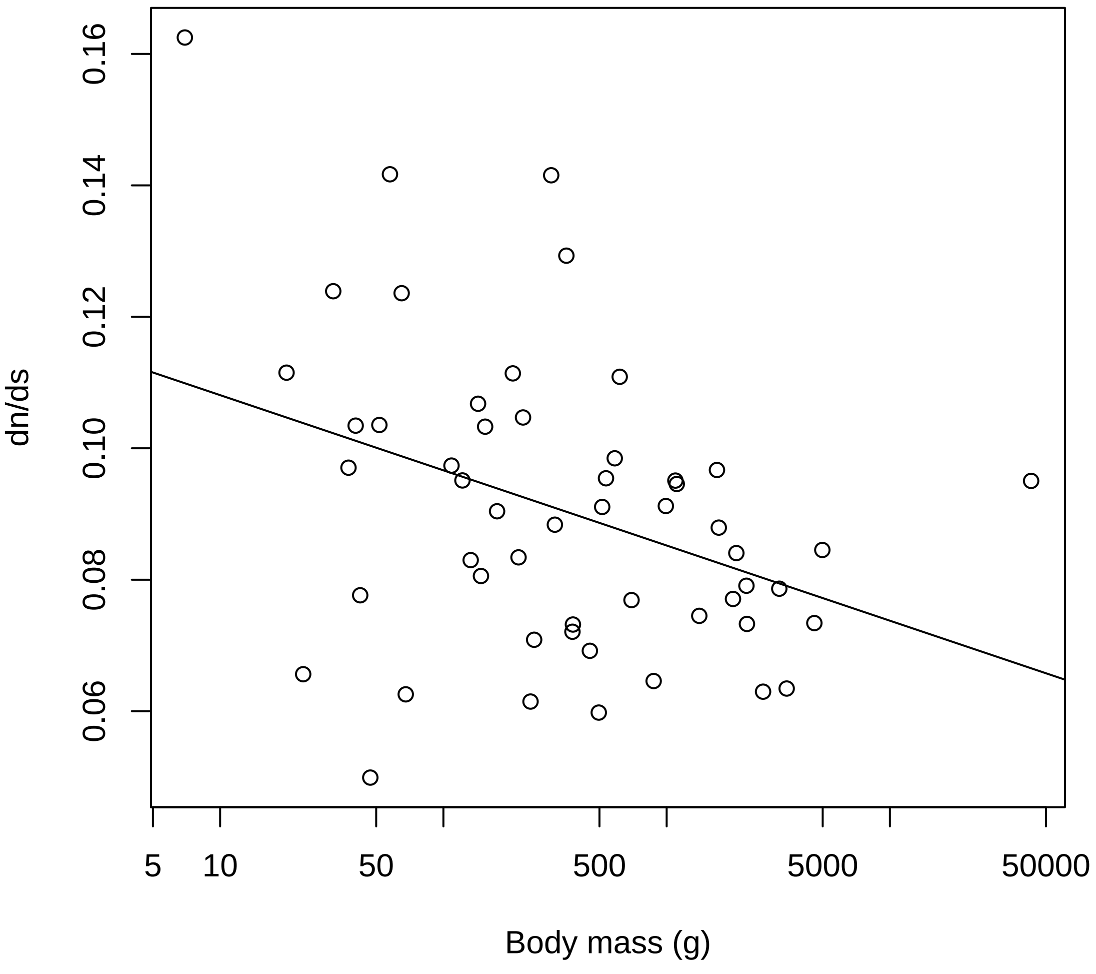

Supplement: Additional file 1 — d N / d S versus mass for the species-rich set. Supplementary information. [file 13059_2014_542_MOESM1_ESM.pdf]
